# The Effects of the Hunterian Method of Ligation on Inflammation

**Published:** 1864-11

**Authors:** Daniel F. Wright

**Affiliations:** Surgeon P. A. C. S.


### CONFEDERATE STATES MEDICAL & SURGICAL JOURNAL.

VOL. I. RICHMOND, NOVEMBER, 1864. No. 11.

### ORIGINAL COMMUNICATIONS.

ART. I.—*The Effects of the Hunterian Method of Ligation on Inflammation.*—By DANIEL F. WRIGHT, Surgeon P. A. C. S.

The observations which I have to make upon the following cases have been mainly suggested by some remarks contained in the third chapter of the Manual of Military Surgery, prepared for the use of the Confederate States Army, by order of the Surgeon-General.

I presume that I am violating no confidence in attributing these remarks to my old and esteemed friend, Surgeon Henry F. Campbell, formerly Professor of Anatomy in the Medical Department of the University of Georgia; they relate to "the *incidental* benefit obtained" by tying the artery above the seat of lesion, as advised by Hunter in preference to Mr. Guthrie's method of ligating in the wound. "The idea pursued," he adds, "in departing from the rule (Guthrie's) was no less than the experimental effort to cure the inflammation in the limb, by cutting off its arterial supply by ligation of the main trunk, which supported that inflammation."

The therapeutic indications aimed at and obtained in three cases specified were—1st, the reduction of excessive œdema; 2d, the amelioration of discharges which changed from sero-sanguinolent to purulent; 3d, the rapid promotion of granulation and cicatrization; and, 4th, in one case the arrest of destructive gangrene. These results are represented to have followed so promptly upon the operation, that with a high degree of probability we are justified in inferring that the ligation was the cause of the improvement. At least a *prima facie* case is abundantly established for further observation, and having been recently placed in a position where the ligation of arteries was frequently called for by the occurrence of secondary hemorrhage, I considered it my duty to notice carefully the changes in the condition of gunshot wounds, subsequent to the ligation of the arteries supplying the part, and to report the results, if they should be such as to throw any light upon the question proposed for consideration. They have in every instance been so entirely in accordance with those obtained in the experiments of Surgeon Campbell, that I cannot feel myself justified in withholding them from publication.

[For the history of the two first I am indebted to Dr. O'Leary, the main incidents having transpired before I was placed in charge of the Division.]

*Case First.*—Winder Hospital, Second Division.—(Service of Assistant-Surgeon O'Leary.)

R. W. Bullet, company "H," Twenty-Second North Carolina regiment; admitted May 24, 1864; wounded May 23, at Hanover Junction. Gunshot wound right foot—point of entrance below internal malleolus, exit at posterior border of *os calcis*. Patient reports that he bled profusely on the field; slight hemorrhage occurred shortly after admission. June 3d—Profuse hemorrhage from the wound; *wound had been cauterized on account of incipient gangrene, and was very unhealthy in appearance;* artery ligated at the seat of injury, and some spiculæ of bone which were exposed by the incision removed from the wound. The appearance of the wound commenced improving from this time. July 3d—Treatment suspended, the wound being healed. July 23d—An abscess in the heel was opened and the man furloughed.

*Case Second.*—Private C. O. Ward, company "G," Sixty-Sixth North Carolina regiment, aged twenty-five. Admitted July 1st, 1864. (Service of Assistant-Surgeon O'Leary.)

Gunshot wound by Minie ball through middle of left hand, fracturing the metacarpal bones of middle and ring finger. June 30—Wound received. July 10—Sloughing and hemorrhage from the palmar arch by plugging the wound with lint saturated with sol. persulph. ferri.; bandaging and elevation of the arm; *sloughing continued and unhealthy action of the wound increased till July* 16—Hemorrhage recurred; compression of radical and ulnar artery failed to arrest it; ligation of brachial artery; condition of the wound commenced improving. July 26—Slight hemorrhage, arrested by cold applications, elevating and bandaging the arm above the elbow. August 1—Abscess on palm opened; spiculæ removed; ordered tinc. ferri. chlor., gutt xx ter quotidie. Aug. 25—Ligature came away. Sept. 6—Furloughed; had servicable motion of thumb, index and little finger.

The following cases occurred under my own observation:

*Case Third.*—Private M. Check, company "I," Sixty-First North Carolina regiment. (Service of Assistant-Surgeon Muldro.)

Flesh wound, with Minie ball entering at about the centre of rectus femoris, exit at the antero interior border of vastus internus, (just over the tract of the femoral artery at lower third of the thigh.) July 30—Wound received at two, P. M. August 3—Profuse hemorrhage from the wound about nine, A. M. My attention was first called to this wound this morning, shortly after the hemorrhage had been checked by Assistant-Surgeon Muldro, by applying a compress firmly over the wound. I determined to leave these dressings on till three, P. M., with but faint hope of their permanently controlling the hemorrhage, as there was every reason to suppose that the hemorrhage came from the femoral artery. At three, P. M., I first applied the tourniquet over the track of the femoral, just above where it crosses the *sartorius* muscle, and then removed the dressings. The condition of the wound was very unhealthy, the edges ragged and apparently sloughing, the tissues discolored and the cavity filled with a dark-colored fetid fluid, the characteristic smell of gangrene exhaling as soon as the wound was uncovered. No pulsation could be felt in the wound or along the track of the femoral, from the wound upwards to the point pressed upon by the tourniquet. I now ordered the tourniquet to be gradually relaxed, and finally taken off, while I watched the results in the wound. No renewal of hemorrhage occurred, though I waited several minutes; again felt along the arterial track, and found that no pulsation could be felt even in the groin, while, on the uninjured side, pulsation was easily felt all along the track, and in the groin very vigorous. Obliteration of the artery for so long a space being very unlikely, the only other supposition was that circulation was suppressed by contraction of the arterial muscular coats. As this could not be calculated upon as permanent, I determined to ligate above the point where the artery divides. That I might see my way thoroughly in this rather problematical condition of things, I made an incision extending down to the wound from a point about three inches above it. (The wound of exit being the one immediately over the artery, of course, was the one selected.) For two inches of this length and throughout the extent of the wound itself the incision was carried down to the artery; in the wound the artery had been torn entirely asunder by the missile; the connective tissue throughout had been so completely destroyed by the diseased action since the injury, that the artery, from its point of division to that selected for ligation, about an inch and a half in length, was separated from surrounding tissues, vessels, nerves, &c., by the fingers alone, without the use of the scalpel. The artery, then, was ligated one inch and a half above the point at which the ball had divided it; the wound was not closed at all, either by sutures or adhesive straps, as the discharges from the wound were at that time so unhealthy that it was deemed that a free escape for them was a more important object than the bringing in apposition parts which in that condition could not unite. My prognosis in the case was far from sanguine. August 4—Fetid discharges diminished; discolored patches disappeared. Aug. 5—Discharge disappeared, wound clean, slight granulations in incision. August 6—Healthy pus discharged; granulations evidently in progress; pulse and general appearance much improved. August 7—Pulsation perceptible in the groin for the first time since the day of operation * * * * * Aug. 13—Ligature came away, the wound entirely filled up with granulations, and cicatrization rapidly advancing. The patient was furloughed about two weeks afterwards, entirely cured.

Deferring for the present any remarks bearing upon the general purport of this paper, I would call attention to the extent over which contraction of the arterial coats took place during the intervals of hemorrhage. I omitted to state that the man reports himself to have fainted from profuse hemorrhage upon the field, as may well be supposed when so large a trunk was divided; nevertheless, the spontaneous contraction of the artery preserved him from further hemorrhage till four days afterwards, (Aug. 3;) and again, after this second hemorrhage had been arrested, the contraction was resumed and extended so far upwards that pulsation could not be felt in the groin—this condition persisting also for four days, (till August 7,) at which time beyond a doubt hemorrhage would have recurred, had not the ligature been applied. A curious instance of the rythmical or periodic type of all intermittent action.

*Case Fourth.*—Sergeant P. Broom, company "A," Forty-Eighth North Carolina regiment. (Service of Assistant-Surgeon Bagnall.)

The middle finger of left hand had been shot away by a Minie ball on the 25th of August. I have not been able to ascertain the exact amount of injury, as amputation had been performed on the field by Surgeon Montgomery, the operation including the excision of the head and some portion of the shaft of the metacarpal bone. As far as I could judge by the appearance of the stump the operation had been skillfully performed. The patient was admitted on the evening of August 26th. The wound upon the whole looked well, except that the incised surfaces were rather dryer than usual in such cases. August 29—Secondary hemorrhage occurred, which was controlled by forcibly flexing the forearm on the humerus, and forcibly retaining it in that position by bandages. Aug. 30—Hemorrhage recurred in the morning; it was temporarily controlled by pressure on the radical and ulnar artery in the wrist, and then the same treatment adopted on the day before; hemorrhage, however, recurred in the afternoon, when ligation was resolved upon. As the bleeding was evidently from the palmar arch, and therefore fed by all three of the main arteries of the forearm, it was concluded to ligate the brachial artery at the lowest point easily accessible. Previously to this, however, the wound was opened. It was found to be filled with a recent coagulum, and when this was removed, there was seen to be a complete absence of all reparative process, either by granulation or adhesion, and a considerable portion of the ulnar lip was effected by dry gangrene, which extended deep into the interior of the divided tissues. The ligature was then applied and the wound closed again. Sept. 2—Some suppuration had commenced, and the lips of the wound looked much healthier. Sept. 3—Suppuration increased. Sept. 4—Phlegmonous erysipelas attacked the arm above the seat of ligation, the progress of which was very rapid, soon producing extensive sloughing of the integuments. The patient was now removed to the tents set apart for the treatment of gangrene and erysipelas, which were not under my charge, and I was unable to see him so frequently as before, but under the judicious treatment of Assistant-Surgeon Meek, the sloughing process was gradually arrested, and (Sept. 18) ligature separated. Sept. 20—All the injured parts are rapidly improving, the integuments in the process of reproduction, and the original wound more than half repaired.

These are all the cases which have occurred under my cognizance since entering upon my present duties, in which the condition of the wound previously to ligation was in such a condition as to fully test the question.

The results will be seen at a glance to be in full accordance with those observed by Surgeon Campbell. Tumefaction, the absence of healthy and presence of unhealthy discharges, arrest of the plastic processes essential to reparation, have, immediately upon the performance of ligation, been superseded by general amelioration in all cases; healthy suppuration, active granulation and spontaneous adhesion of apposed surfaces have rapidly issued in the healing of the wounds, except in the last case, in which the reparative process was indeed commenced, but arrested by the intercurrent erysipelas.

In none of these cases was ligation resorted to with the direct purpose of reversing the morbid action; but while it was adopted for the purpose, in each case, of arresting hemorrhage, its influence upon the pathological condition of the injured case was so marked in promoting the desired reparative action, that I cannot but conclude that a case is fairly established for resorting to it for its direct beneficial effects, apart from the necessity of checking the bleeding which frequently occurs in wounds of the morbid character described. The first ligation of a large artery, not necessitated by hemorrhage, will be a bold experiment, but if the occasion be well chosen I think that the results will justify the daring, and the expedient will become an established procedure in surgical art.

